# Chemotherapeutics Used for High-Risk Neuroblastoma Therapy Improve the Efficacy of Anti-GD2 Antibody Dinutuximab Beta in Preclinical Spheroid Models

**DOI:** 10.3390/cancers15030904

**Published:** 2023-01-31

**Authors:** Sascha Troschke-Meurer, Maxi Zumpe, Lena Meißner, Nikolai Siebert, Piotr Grabarczyk, Hannes Forkel, Claudia Maletzki, Sander Bekeschus, Holger N. Lode

**Affiliations:** 1 Department of Pediatric Oncology and Hematology, University Medicine Greifswald, Ferdinand-Sauerbruch Strasse 1, 17475 Greifswald, Germany; 2 Department of Internal Medicine, Clinic III—Hematology, Oncology, University Medicine Greifswald, Ferdinand-Sauerbruch Strasse 1, 17475 Greifswald, Germany; 3 Department of Medicine, Clinic III—Hematology, Oncology, Palliative Medicine, Rostock University Medical Center, Ernst-Heydemann-Str. 6, 18057 Rostock, Germany; 4 ZIK Plasmatis, Leibniz Institute for Plasma Science and Technology (INP), Felix-Hausdorff-Str. 2, 17489 Greifswald, Germany

**Keywords:** ADCC, carboplatin, chemoimmunotherapy, cisplatin, cyclophosphamide, dinutuximab beta, etoposide, neuroblastoma, vincristine

## Abstract

**Simple Summary:**

We investigated the effects of chemotherapeutics used for the frontline treatment of newly diagnosed high-risk neuroblastoma patients in combination with anti-GD2 antibody ch14.18/CHO (dinutuximab beta, DB) in the presence of immune cells in preclinical models of neuroblastoma. The combined treatment showed an up-to-17-fold-stronger and GD2-specific cytotoxic effect compared to the controls treated with chemotherapy alone in the presence or absence of immune cells. These findings further support a clinical evaluation of DB in combination with frontline induction therapy for high-risk neuroblastoma patients.

**Abstract:**

Anti-disialoganglioside GD2 antibody ch14.18/CHO (dinutuximab beta, DB) improved the outcome of patients with high-risk neuroblastoma (HR-NB) in the maintenance phase. We investigated chemotherapeutic compounds used in newly diagnosed patients in combination with DB. Vincristine, etoposide, carboplatin, cisplatin, and cyclophosphamide, as well as DB, were used at concentrations achieved in pediatric clinical trials. The effects on stress ligand and checkpoint expression by neuroblastoma cells and on activation receptors of NK cells were determined by using flow cytometry. NK-cell activity was measured with a CD107a/IFN-γ assay. Long-term cytotoxicity was analyzed in three spheroid models derived from GD2-positive neuroblastoma cell lines (LAN-1, CHLA 20, and CHLA 136) expressing a fluorescent near-infrared protein. Chemotherapeutics combined with DB in the presence of immune cells improved cytotoxic efficacy up to 17-fold compared to in the controls, and the effect was GD2-specific. The activating stress and inhibitory checkpoint ligands on neuroblastoma cells were upregulated by the chemotherapeutics up to 9- and 5-fold, respectively, and activation receptors on NK cells were not affected. The CD107a/IFN-γ assay revealed no additional activation of NK cells by the chemotherapeutics. The synergistic effect of DB with chemotherapeutics seems primarily attributed to the combined toxicity of antibody-dependent cellular cytotoxicity and chemotherapy, which supports further clinical evaluation in frontline induction therapy.

## 1. Introduction

Neuroblastoma is the leading cancer-related cause of death in children [1]. Despite intensive multimodal treatment options, the long-term event-free survival is still only 50% [2]. Neuroblastoma cells highly express the tumor-associated antigen disialoganglioside GD2, which can be targeted with the monoclonal antibody (mAb) dinutuximab beta (DB, ch14.18/CHO). Although this therapy has increased the overall survival of patients (pts) with high-risk neuroblastoma (HR-NB) at 5 years by 15% [3], new treatment options are needed to further improve the outcome.

One promising approach is the combination of antibody treatment with chemotherapy. In a prospective randomized trial conducted by the Children’s Oncology Group (COG) in patients with relapsed or refractory neuroblastoma, pts treated with dinutuximab (ch14.18/SP2/0) combined with irinotecan, temozolomide, and granulocyte-macrophage stimulating factor (GM-CSF) showed an objective response rate of 41.5% [4]. In a non-randomized study, anti-GD2 antibody hu14.18K322A was combined with six cycles of the COG induction chemotherapy and granulocyte-macrophage colony-stimulating factor and low-dose interleukin-2 (IL-2) in newly diagnosed high-risk neuroblastoma patients [5]. The end-of-induction partial response and complete response rate were 97%, and no patients experienced progressive disease during induction, suggesting an improvement over historical control.

However, the use of the anti-GD2 antibody DB during the European neuroblastoma chemotherapy induction regimen has not been evaluated yet [6]. The main mechanism of action of a DB-based immunotherapy is the induction of antibody-dependent cellular cytotoxicity (ADCC), and the combination of such chemotherapeutics with DB might have differential effects on antitumor efficacy.

Chemotherapeutics kill tumor cells by means of genotoxic stress or prevention of mitosis, resulting in apoptosis, senescence, and immunogenic cell death.

Studies showed that the mere reduction of the tumor cell mass during chemotherapy improves the immunological antitumor response [7]. Additionally, an increasing body of evidence suggests that an innate immune response is crucial for the antitumor activity of chemotherapeutics [8,9,10], as they induce immunogenic cell death that can increase antigen presentation and elicit a cytotoxic T-cell response [11]. In this context, the PD-1 immune checkpoint blockade improved CD8+ T-cell effector functions during chemotherapy [12].

Immunological advantages of chemotherapy also emerge from an increased visibility of tumor cells to the immune system mediated by stress ligand expression, and NK cells are the main effector cells that kill tumor cells upon stress-ligand recognition [13,14]. Stress ligands can bind NK-cell-specific activating receptors such as NKp30 (receptor for B7-H6) and NKG2D (receptor for ULBPs and MICA/B) and therefore tip the balance toward NK-cell stimulation [15]. Accordingly, it has been shown that chemotherapy-induced B7-H6 sensitizes leukemia and solid tumor cells for NK-cell-mediated cytotoxicity [16].

Importantly, agents used in European neuroblastoma induction regimens rapid COJEC and GPOH [6,17], such as cisplatin and vincristine, induced B7-H6 and ULBP expression in cell lines, e.g., multiple myeloma and human embryonic kidney cells (HEK293) [13,16]. In line with that, the combination treatment of cisplatin and NK cells has proven to overcome the chemotherapy resistance of cancer cells by inducing ULBP stress ligands in vitro [18]. Despite the fact that chemotherapeutics have been considered to be immune-inhibitory agents, there is evidence that NK cells are functional during chemotherapy [19]. Therefore, chemoimmunotherapy might be an excellent tool for enhancing antitumor efficacy during induction therapy in HR-NB.

However, the beneficial effects of chemotherapeutics combined with the antitumor effects of therapeutic antibodies (ADCC) may be counter-regulated by inducing immune checkpoints on tumor cells. We and others have shown that ADCC and also chemotherapies induce PD-L1 expression, leading to inhibited antibody-mediated tumor killing [20,21]. A chemoimmunotherapy therefore might be hindered by tumor cells harnessing immune checkpoint pathways to escape immune surveillance [18].

Due to the mode of action of chemotherapies, a long-term analysis of antitumor activity of chemotherapeutics is imperative [22]. We therefore established a live-cell neuroblastoma spheroid model to assess long-term chemotherapeutic effects. A spheroid model is a 3-dimensional (3D) spherical aggregation of tumor cells that represent a complex tumor environment and architecture including zones of proliferation at the outside and quiescent cells in the inside [23,24,25]. Therefore, spheroid models provide a more clinically relevant model regarding chemotherapy diffusion, angiogenesis, tumor invasion and chemotherapy resistance compared to 2D models [23,26,27,28,29,30].

We used the described spheroid model, to test the hypothesis that combining chemotherapeutic agents used in induction therapy regimens for pts with HR-NB with DB can enhance antitumor efficacy and determined their effect on activating and inhibitory receptors and ligands on target and effector cells.

## 2. Materials and Methods

### 2.1. Cell Culture

The human NB cell line LA-N-1 was cultured in RPMI (PAN BIOTECH, P04–016520) supplemented with 4.5 g/L glucose, 2 mM stable glutamine, 10% FCS and 100 U/mL penicillin, and 0.1 mg/mL streptomycin (1× P/S; PAN BIO- 485 TECH, P06–07100). The human NB cell lines CHLA-20 and CHLA-136 were cultured in IMDM (PAN BIOTECH, P04–20250) supplemented with 4 mM stable glutamine, 20% FCS, 1× ITS (BD Biosciences, 3220669), and 1× P/S. Human PBMCs (peripheral blood mononuclear cells) were isolated from whole blood concentrates without serum of healthy donors, using the Pancoll separating method (human, density 1.077 g/mL, BIOTECH, P04-60500). PBMCs were cultured in RPMI supplemented with 10% FCS, 50 µM, 100 IU/mL, IL-2, β-mercaptoethanol, and 1× P/S for 72 h prior to the experiments. 

### 2.2. Chemotherapy and Antibodies for Cytotoxicity Assay

Tumor cells were treated with drug concentrations of carboplatin (2 µg/mL), cisplatin (1 µg/mL), etoposide (0.1–1 µg/mL), vincristine (0.05 µg/mL), and 4-HPC (2 µg/mL), as achieved in pediatric pharmacokinetic studies [5,31,32,33,34,35]. Cells were washed 24 h after the start of treatment. Solutions of chemotherapeutics were produced by the university pharmacy Greifswald and used within 28 days. The antibody DB was used at a concentration of 10 µg/mL in line with concentrations achieved in clinical trials [36]. DB was purchased from EUSA Pharma (UK), and rituximab from Roche (Switzerland).

### 2.3. Stable Transduction of Tumor Cells Using Lentiviral Vectors

For recombinant lentivirus production, a second-generation lentiviral vector system was used. The non-confluent Lenti-X™ 293T cells were co-transfected with purified pVSV-G-envelope-expressing plasmid (Addgene, Watertown, MA, USA), psPAX2 (Addgene, Watertown, MA, USA) vector encoding virus polymerase and packaging genes, and lentiviral vector pWPXL (Addgene, Watertown, MA, USA) coding for a near-infrared reporter (NIR) (iRFP680). The transfection of Lenti-X™ 293T cells was conducted by using CalPhos Mammalian Transfection Kit (Takara Bio Europe, Saint-Germain-en-Laye, France) according to the manufacturer’s instructions. For transduction, 8 mg/mL Polybrene (Merck KGaA, Darmstadt, Germany) and lentiviral supernatants were added to the target cells. Target cells were cultured under cell culture conditions, and after 72 h, they were tested for the successful transduction of the IncuCyte^®^ SX5 live-cell analysis system (Sartorius, Göttingen, Germany).

### 2.4. Long-Term Live-Cell Spheroid Viability Assay and Treatment Conditions

To yield three-dimensional (3D) tumor spheroids, we used ultra-low attachment plates (ULA plates, S-BIO PrimeSurface^®^, MS-90384UZ) that were precoated with a hydrophilic polymer facilitating spontaneous self-assembly by preventing cellular attachment to the surface. This method was recently shown to be the best approach for studying the efficacy of drugs with respect to spheroid maintenance and reproducibility of results [37]. A total of 3000 iRFP680-positive neuroblastoma cells were seeded into ULA 384-well plates. For CHLA-136, we additionally used 0.5% Matrigel to improve spheroid formation. Cells were centrifuged at 150× *g* for 10 min and incubated for 72 h at 37 °C and 5% CO_2_. Spheroids were treated with the respective chemotherapeutic compound for 24 h, followed by the addition of 22,500 PBMCs with and without the anti-GD2 antibody DB and incubation for a further 216 h under cell culture conditions.

Image acquisition was performed every 8 h for 240 h (10 days), using the IncuCyte^®^ SX5 live-cell analysis system. Spheroid viability was calculated as the ratio of integrated spheroid fluorescence intensity of every time point to fluorescence at baseline (0 h). Experiments were performed in six replicates, and viability is reported in %±SEM.

### 2.5. Flow Cytometry

#### 2.5.1. Validation of Near Infrared Reporter (NIR) as Viability Marker

To validate NIR fluorescence (680 nm) from stable transduced NB tumor cells as the viability marker, flow cytometry analyses were performed by using 40,6-diamino-2-phenylindole (DAPI) (Merck KGaA, Darmstadt, Germany). For these analyses, 50% of the cells were lysed (65 °C, 5 min) to obtain samples with live and dead cells in an equal amount. Next, cells were incubated with a 0.1 mg/mL DAPI solution (Sigma-Aldrich, D9542) for 5 min prior to acquisition. For each sample, 20,000 cells were analyzed by using a BD CANTO II cytometer and FACS Diva software (BD Biosciences, San Jose, CA, USA). Data were analyzed with FlowJo V10 software (Ashland, OR, USA) analyzing the frequency of NIR- and DAPI-positive and -negative cells of all single cells.

#### 2.5.2. Stress-Ligand Abundance on Chemotherapy-Treated Tumor Cells

For the analysis of the stress ligand abundance of chemotherapy-treated tumor cells, 1 × 10^6^ live neuroblastoma cells were seeded into Petri dishes in 10 mL of respective medium and cultured for one day (37 °C/5% CO_2_). Cells were treated with chemotherapy as described above and washed after 24 h. After 72 h, 1 × 10^6^ live cells were washed and treated with Zombie NIR™ Fixable Viability Dye (Biolegend, RT, San Diego, CA, USA) according to the manufactures protocol. The cells were then incubated with the following antibodies: anti-human B7-H6-APC (mouse IgG1, clone 875001), ULBP-1-PerCP (mouse IgG2a, clone 170818), ULBP-2/5/6-Alexa Fluor^®^ 405 (mouse IgG2a, clone 165903), ULBP-3-PE (mouse IgG2a, clone 166510) all from R & D Systems, 1:20 diluted, and MICA/MICB-PE-Cy7 (Biolegend, mouse IgG2a,κ, clone 6D4, 1:20). A total of 20,000 live cells were measured per sample. Due to chemotherapy-related changes of the autofluorescence, we determined the expression level of respective antigen according to the following formula: MFI of stained sample—MFI of unstained sample.

#### 2.5.3. Immune Checkpoint Ligand Abundance on Chemotherapy-Treated Tumor Cells

Immune checkpoint ligand expression analysis by tumor cells was performed in analogy to the stress ligand expression analysis detailed above, using the following antibodies: anti-human CD80-BV421 (Biolegend, mouse IgG1,κ, clone 2D10, 1:20), CD86-PerCP-Vio 700 (Miltenyi Biotec, Bergisch Gladbach, REA968, 1:50), CD112-APC (Miltenyi Biotec, REA1195, 1:50), CD155-PE-Vio 770 (Miltenyi Biotec, REA1081, 1:50), Galectin-9-PE (Miltenyi Biotec, REA435, 1:50), and CD274 (PD-L1)-Vio Bright B515 (Miltenyi Biotec, REA1197, 1:50). The mouse IgG1,κ antibody-BV421 (Biolegend, clone MOPC-21) and respective REA controls (Miltenyi Biotec, REA293) were used as isotype controls.

#### 2.5.4. NK Cell Activation after Chemotherapy Treatment

For the analysis of activating receptor expression by cytotoxic NK cells (CD3-; CD56dim), 5 × 10^6^ human PBMCs were treated with the respective chemotherapeutic compound and incubated for 72 h (37 °C/5% CO_2_). Then cells were harvested and 1 × 10^6^ live cells were washed with wash buffer, followed by incubation with 10 µL of Tandem Signal Enhancer (Miltenyi Biotec). Incubation with the following antibodies in a total volume of 100 µL was conducted for 20 min at RT: CD3-VioGreen (REA613, 1:200), CD56-APC-Vio770 (REA196, 1:200), CD226-VioBlue (REA1040, 1:50); CD335 (NKp46)-Vio Bright B515 (REA808, 1:50), CD337 (NKp30)-PE (REA823, 1:75), CD336 (NKp44)-APC (REA1163, 1:75), and CD314 (NKG2D)-PE-Vio 770 (REA1228, 1:75), all from Miltenyi Biotec. As isotype controls served respective REA controls (Miltenyi Biotec, REA293). To exclude dead cells from analysis, 0.25 µg propidium iodide solution was added prior to acquisition. At least 20,000 CD3−/CD56+ cells were analyzed for each sample.

#### 2.5.5. CD107a Degranulation Assay

First, 0.25 × 10^6^ tumor cells LAN-1 and CHLA-20 and respective B7-H6 knockout cells generated as described below were seeded into a 24-well plate in 1 mL RPMI. After 24 h, cells were treated with etoposide (LAN-1) and carboplatin (CHLA-20) and washed after 24 h. After a further 48 h, medium was removed, and 2 × 10^6^ PBMCs, including 2 µL Brefeldin A (Invitrogen, Waltham, MA, USA), 2 µL Monensin (Invitrogen), 1 µg/µL DB, and 2 µL CD107a-VBV515 (Miltenyi Biotec, REA792) in a total volume of 1 mL RPMI, were added. PBMCs without tumor cells and DB served as the control. Cells were incubated for 5 h under cell culture conditions. Then cells were stained by using Viobility™ 405/452 Fixable Dye (1:100 in 1× PBS, 15 min). Cells were fixed by using Inside Stain Kit (Miltenyi Biotec, 130-090-477) according to manufacturer’s guidelines. A total of 10 µL of Tandem Signal Enhancer was added prior to cell-surface staining with CD3-VioGreen (REA613, 1:200), CD16-PerCP-Vio770 (REA423, 1:50), and CD56-APC-Vio770 (REA196, 1:200), all from Miltenyi Biotec and CD45-PE-Cy7 (BD Biosciences, mouse IgG1, clone J33, 1:300). After permeabilization, using an Inside Stain Kit, intracellular staining with INF-γ-APC (REA600, 1:50, 100 µL, 20 min) was performed. At least 20,000 CD45+/CD3−/CD56+ cells were analyzed for each sample.

### 2.6. CRISPR/CAS9 B7-H6 Knockout in Tumor Cells

To delete B7-H6 (NCR3LG1 locus), 1 × 10^6^ tumor cells were transfected by using SF Cell Line Solution (Lonza, Switzerland) and program FF-120. Following DNA target sequences of the NCR3LG1, crRNAs were used: 5′-GTGTGTGGTACGGCATGCGT-3′, 5′- TCACGTCTATGGGTATCACC-3′, and 5′-CACCAAGAGGCATTCCGACC-3′. Successful abrogation of B7-H6 was shown via flow cytometry analysis and stable deletion was confirmed regularly (every four weeks).

### 2.7. Statistics

Differences between the groups were assessed by using ANOVA with Dunnett’s post hoc test, if the assumption of normality was met (Shapiro–Wilk Test). Due to donor-dependent variability of PBMCs-dependent antitumor toxicity, the significant difference between the groups was analyzed by using repeated measurement ANOVA for individual data points. Statistical analysis was performed by using GraphPad Prism (version 9.4.1 for Windows, GraphPad Software, San Diego, CA, USA). Viability data are presented as mean ± SEM (standard error of the mean), and flow cytometry data are shown as individual data point with mean and SEM indicated.

## 3. Results

### 3.1. Establishment of a Long-Term Real-Time Viability Assay

**Figure 1:**
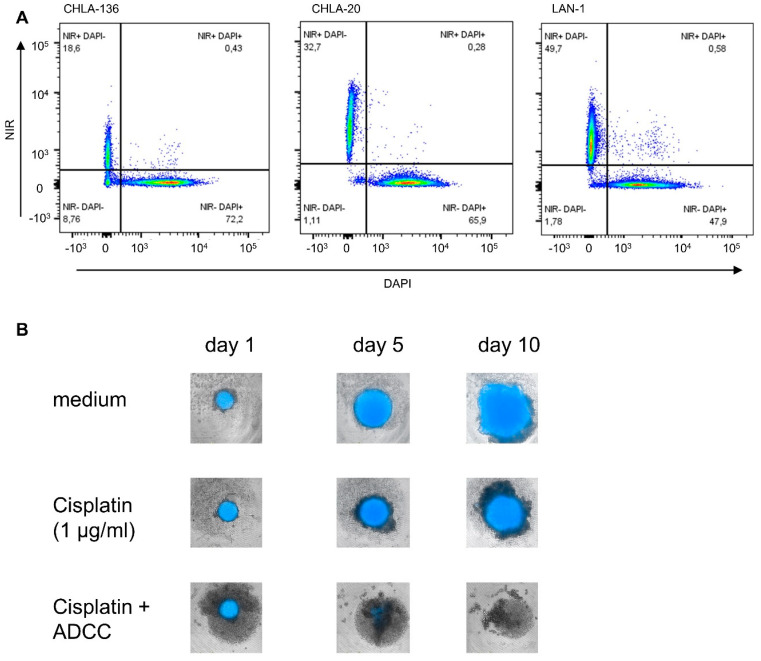
Establishment of a live-cell assay for analysis of long-term chemoimmunotherapeutic effects on tumor cell viability. (**A**) Flow cytometric analysis of neuroblastoma cells transduced to yield NIR-fluorescence (680 nm) used as viability marker. NIR+ cells were lysed, mixed with living cells, and subsequently stained with DAPI to confirm the correct discrimination of live and dead cells, using the NIR fluorescence. About 99.5% of live and dead cells could be correctly identified by using NIR-fluorescence: NIR-positive cells (live) were DAPI-negative, and NIR-negative cells (dead) were DAPI-positive. (**B**) Representative picture showing loss or gain of spheroid fluorescence intensity under therapy.

**Figure 2:**
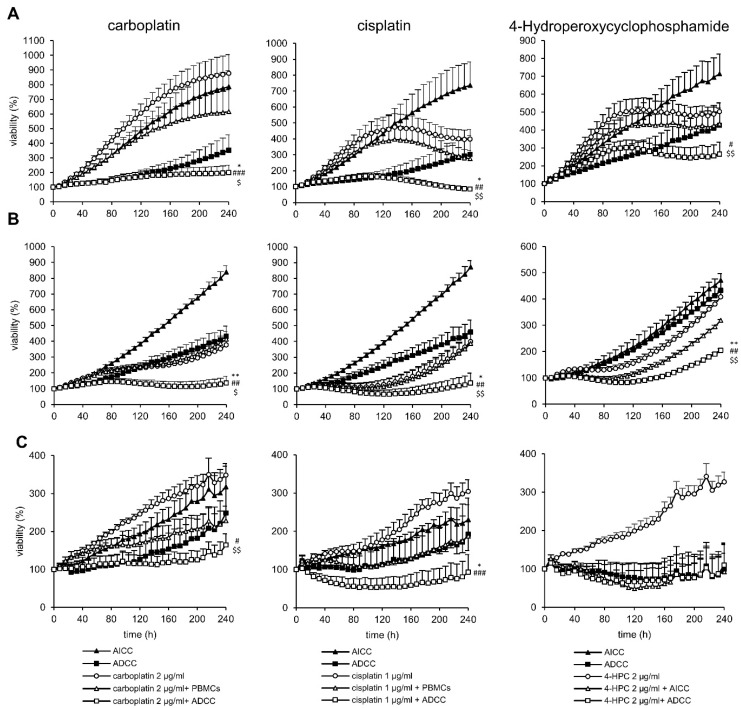
Impact of 2 µg/mL carboplatin and 1 µg/mL cisplatin and cyclophosphamide (4-Hydroperoxycyclophosphamide) on ADCC against the chemotherapy resistant cell line (**A**) LAN-1, (**B**) CHLA-20, and (**C**) CHLA-136. Tumor cells were transduced with a gene coding for near-infrared fluorescent protein (iRFP680) to track viability. To establish stable spheroids, 3000 tumor cells/well were seeded into a 384-well plate and cultivated for three days under cell culture conditions. Spheroids were then treated with their respective chemotherapies, which were removed after 24 h, or PBMCs or the anti-GD2 antibody DB and incubated for a further seven days. Viability was calculated as total integrated spheroid fluorescence of the respective time point divided by the total fluorescence at time point 0 h. Graphs show viability of spheroids treated with or without chemotherapy (black line, white or black marker, respectively), PBMCs alone (solid triangle), ADCC (solid square). Data are shown as means from four independent experiments (performed in six replicates) ± SEM. Endpoint (240 h). Viability data of ADCC (*), chemotherapy (#), and chemotherapy + AICC ($) vs. chemotherapy + ADCC were compared by using repeated measures ANOVA; ∗∗ *p* < 0.01; ∗ *p* < 0.05 vs. ADCC; ^###^ *p* < 0.001; ^##^ *p* < 0.01; ^#^ *p* < 0.05 vs. chemotherapy; ^$$^ *p* < 0.01; ^$^ *p* < 0.05 vs. chemotherapy + ADCC.

We developed a long-term viability assay by using live-cell image acquisition and fluorescent tumor cells. First, the neuroblastoma cell lines LAN-1, CHLA-136, and CHLA-20 were transduced by using a lentiviral expression system to yield stable expression of the fluorescent near-infrared protein iRFP680 (NIR) used as viability staining [38]. Stable expression was confirmed by flow cytometry up to one month after transduction. Only cell lines with over 95% NIR^+^ cells were used. The correlation between viability and NIR-fluorescence status was confirmed with DAPI staining and analyzed by flow cytometry (Figure 1). NIR-fluorescence was an accurate marker for viability in over 95% of tumor cells analyzed (99.3%, 99.6%, and 97.6% were NIR+ and DAPI- or NIR- and DAPI+, for LAN-1, CHLA-20, and CHLA-136, respectively) (Figure 1A). Importantly, we found that the effects of chemotherapeutics used in concentrations realistic in the clinical setting were only measurable from day four after the start of the treatment, showing the requirement for long-term assays to assess effects (Figure 1B and Figure 2A, cisplatin, Video S1–S3 for cisplatin + ADCC and controls in LAN-1).

### 3.2. Effects of Chemotherapeutics on Antibody-Dependent Cellular Cytotoxicity (ADCC)

We investigated how chemotherapeutics affect the ADCC with DB (10 µg/mL) and effector cells (PBMCs (7.5 × 10^4^ cells) against established tumor spheroids generated from the three cell lines, using real-time viability assay over 10 days.

**Figure 3:**
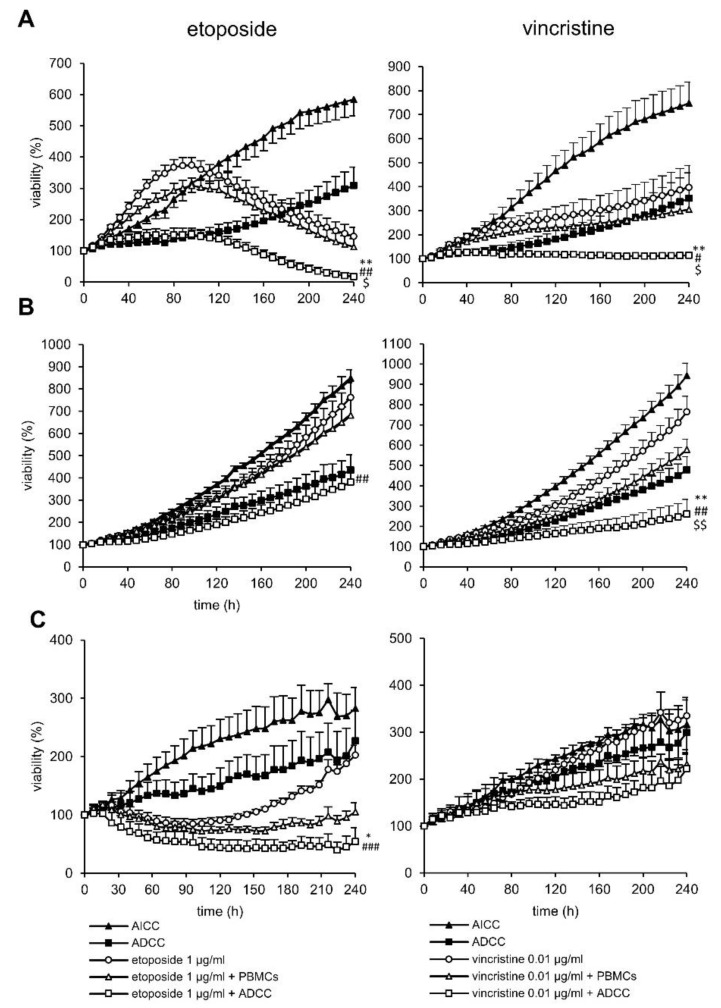
Impact of 0.1 (CHLA-20) −1 µg/mL etoposide (LAN-1 and CHLA-136) and 0,05 µg/mL vincristine on ADCC against the chemotherapy resistant neuroblastoma cells (**A**) LAN-1, (**B**) CHLA-20, and (**C**) CHLA-136. Tumor cells were transduced with a gene coding for near-infrared fluorescent protein (iRFP680) to track viability. To establish stable spheroids, 3000 tumor cells/well were seeded into a 384-well plate and cultivated for three days under cell culture conditions. Spheroids were then treated with respective chemotherapy, which was removed after 24 h, or PBMCs or with the anti-GD2 antibody DB, and incubated for a further seven days. Viability was calculated as total integrated spheroid fluorescence of the respective time point divided by the total fluorescence at time point 0 h. Graphs show viability of spheroids treated with or without chemotherapy (black line, white or black marker, respectively), PBMCs alone (solid triangle), ADCC (solid square). Data are shown as means from four independent experiments ± SEM. Endpoint (240 h). Viability data of ADCC (*), chemotherapy (#), and chemotherapy + AICC ($) vs. chemotherapy + ADCC were compared by using repeated measures ANOVA; ** *p* < 0.01; * *p* < 0.05 vs. ADCC; ^###^ *p* < 0.001; ^##^ *p* < 0.01; ^#^ *p* < 0.05 vs. chemotherapy; ^$$^ *p* < 0.01; ^$^ *p* < 0.05 vs. chemotherapy + ADCC.

The chemotherapeutics used at clinically relevant concentrations combined with DB and effector cells (ADCC condition) had an up-to-17-fold-higher long-term antitumoral effect in this model (*p* = 0.0029). ADCC conditions also showed a delayed tumor growth that was stronger compared to the controls of chemotherapeutics combined with effector cells only (without DB; antibody-independent cellular cytotoxicity (AICC); see Figure 2 and Figure 3). This clearly indicates a DB-dependent and GD2 specific effect. The viability curves of AICC with and without chimeric isotype control (rituximab) were not different.

There was a differential pattern of efficacy depending on the cell line used to establish the spheroids. For instance, the chemoimmunotherapy with platin agents (cisplatin and carboplatin) significantly improved the antitumoral effects compared to chemotherapy alone or compared to chemotherapy with effector cell only (AICC) in LAN-1 and CHLA-20 spheroids, but to a lesser extent in CHLA-136 spheroids (3.6-, 2.8-, and 2.0-fold decrease in viability (at 10 d) versus AICC in combination with cisplatin, respectively; for overview, see Table 1). Cyclophosphamide significantly increased ADCC in LAN-1- and CHLA-20 spheroids but not in CHLA-136 (1.6-, 1.6-, and 0.8-fold decrease in viability compared to AICC + cyclophosphamide, respectively; see Figure 2A, right panel). This might be attributable to higher resistance of CHLA-136 against cyclophosphamide compared to LAN-1 and CHLA-20 (Figure 2C, right panel).

Chemoimmunotherapy with etoposide was highly effective against LAN-1 and CHLA-136 spheroids (6.4- and 2.9-fold decrease in viability compared to AICC in combination with etoposide; see Figure 3A,C), whereas CHLA-20 spheroids were too sensitive to etoposide with PBMCs to allow for a differentiation between chemoimmunotherapy with AICC and ADCC, even at low concentrations of etoposide (0.1 µg/mL, 1.4-fold decrease; see Figure 3B). Chemoimmunotherapy with vincristine was significantly more effective in LAN-1 and CHLA-20 spheroids, but not in CHLA-136-spheroids (Figure 3A–C) (2.7-, 2.2-, and 1.0-fold decrease in viability compared to AICC + vincristine, respectively; see Figure 3A–C, right panel).

In conclusion, chemotherapeutics used in induction regimens combined with DB and effector cells showed superior effects against neuroblastoma spheroids compared to the respective controls.

### 3.3. Chemotherapy-Induced Stress Ligands on Tumor Cells

To further investigate the reasons for the observed improved antitumor effects of chemoimmunotherapy compared to monotherapy controls, we investigated the induction of stress ligands involved in NK-cell activation (B7-H6, ULBP 1–3 and MICA/B) three days after chemotherapy, using flow cytometry.

**Figure 4:**
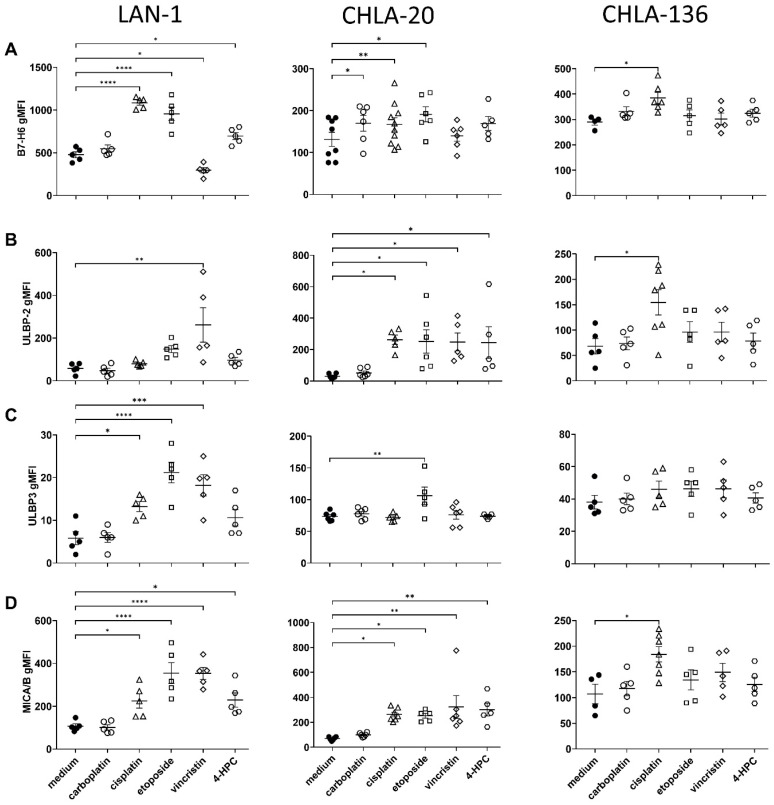
Chemotherapy-induced stress-ligand surface abundance on tumor cells: 1 × 10^6^ tumor cells treated for 24 h under cell culture conditions with either carboplatin (2 µg/mL, open circles), cisplatin (1 µg/mL, open triangles) etoposide (0.5 µg/mL, open squares), vincristine (0.05 µg/mL, open diamonds), or the cyclophosphamide metabolite 4-hydroperoxycyclophosphamide (1 µg/mL, open hexagons). After 72 h of culturing, LAN-1 (left panel), CHLA-20 (center), and CHLA-136 (right panel) were analyzed for surface abundance of (**A**) B7-H6, (**B**) ULBP2, (**C**) ULBP-3, and (**D**) MICA/B, using flow cytometry. Data represent at least five biological replicates. Means and SEM are indicated as black lines and error bars, respectively. For statistical analysis, ANOVA with appropriate post hoc test was used; * *p* < 0.05, ** *p* < 0.01, *** *p* < 0.001, and **** *p* < 0.0001 vs. untreated control (medium).

All cell lines showed a measurable B7-H6 (NKp30 ligand) but low ULBP and MICA/B (NKG2D ligands) baseline cell surface abundance (Figure 4). The pattern of chemotherapy-dependent induction of stress ligands was cell-line specific. In LAN-1, the NKp30 ligand B7-H6 was significantly increased by cisplatin, etoposide, and cyclophosphamide treatment compared to controls (2.3-, 2.0-, and 1.5-fold; *p* < 0.0001, <0.0001, and *p* = 0.0111, respectively), in CHLA-20 by carboplatin, cisplatin, and etoposide (1.2-, 1.2-, and 1.4-fold, *p* = 0.024, 0.0041, and 0.113, respectively) and in CHLA-136 by cisplatin (1.3-fold, *p* = 0.0129) (Figure 4A). This is in line with a higher level of antitumor toxicity by DB, immune cells, and platin compounds compared to the platin compounds and AICC (Figure 2).

We found a differential induction of the NKG2D ligands ULBP2, ULBP-3, and MICA/B in all three cell lines (Figure 4B–D). Interestingly, all chemotherapeutics except carboplatin significantly increased ULBP-2 and MICA/B in CHLA-20 and in LAN-1 (up to 4.5- (vincristine) and 3.3- (etoposide) and up to 9- (cisplatin) and 4.5-fold (cyclophosphamide). We mainly observed effects on ULBP3 abundance after cisplatin, etoposide, and vincristine treatment (up to 3.6-fold increase).

Overall, most chemotherapeutics elicited a stress response in LAN-1 and in CHLA-20. However, only cisplatin significantly affected CHLA-136 stress-ligand surface abundance (B7-H6, ULBP-2, and MICA/B, up to 2.3-fold increase).

### 3.4. Chemotherapy Increased Immune Checkpoint Ligand Surface Abundance

**Figure 5:**
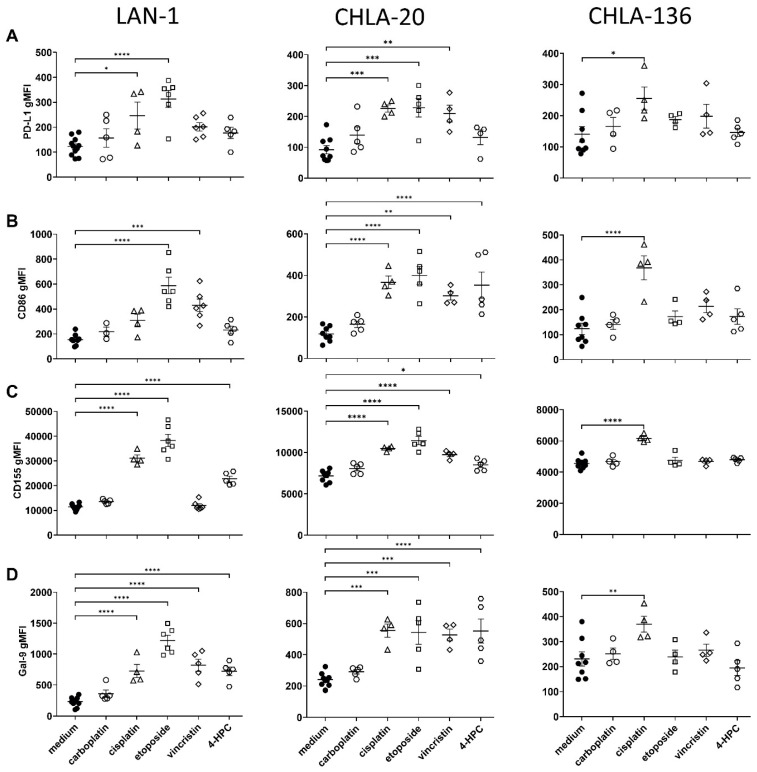
Chemotherapy-induced immune-checkpoint ligand-cell surface abundance on tumor cells. 1 × 10^6^ tumor cells treated for 24 h under cell culture conditions with either carboplatin (2 µg/mL, open circles), cisplatin (1 µg/mL, open triangles), etoposide (0.5 µg/mL, open squares), vincristine (0.05 µg/mL, open diamonds), or the cyclophosphamide metabolite 4-HPC (1 µg/mL, open hexagons). After 72 h of culturing, LAN-1 (left panel), CHLA-20 (center), and CHLA-136 (right panel) were analyzed for surface abundance of (**A**) PD-L1, (**B**) CD86, (**C**) CD155, and (**D**) Gal-9, using flow cytometry. Data represent at least five biological replicates. Means and SEM are indicated as black lines and error bars, respectively. For statistical analysis, ANOVA with appropriate post hoc test was used; * *p* < 0.05 vs., ** *p* < 0.01, *** *p* < 0.001, and **** *p* < 0.0001 versus untreated control (medium).

We investigated chemotherapy effects on the expression of PD-L1 (PD-1), CD86 (CTLA-4), CD155 (TIGIT), and Gal-9 (TIM-3) immune checkpoint ligands by neuroblastoma cells (Figure 5). In LAN-1, etoposide showed the strongest effects on all checkpoint ligands analyzed (2.6-, 4.1-, 3.3-, and 5.2-fold increase for PD-L1, CD86, CD155, and Gal-9, *p* < 0.0001 respectively). Cisplatin had a significant impact on PD-L1, CD155, and Gal-9 but not on CD86 (2.3-, 2.7-, and 3.1-fold increase; *p* = 0.0115, *p* < 0.0001, and *p* = 0.0998), vincristine significantly elevated CD86 and Gal9 expression (3.0- and 3.3-fold increase; *p* = 0.0002 and *p* < 0.0001), whereas cyclophosphamide increased the expression of CD155 and Gal-9 (2.1- and 3.1-fold increase, *p* < 0.0001).

CHLA-20 cells also revealed a strong induction of immune checkpoints by all chemotherapeutics, except for carboplatin (up to 2.4-fold increase, cisplatin, Gal-9, *p* = 0.0002; see Figure 5A–D). Interestingly, and in line with the stress-ligand results, we only observed a cisplatin-dependent rise in PD-L1, CD86, CD155, and Gal9 surface abundance on CHLA-136 tumor cells (1.7-, 2.8-, 1.6-, and 1.3-fold increase; *p* = 0.0222, *p* < 0.0001, *p* < 0.0001, and *p* = 0.0093, respectively).

These data indicate that checkpoint ligand expression also correlates with the tumor stress response following chemotherapy.

### 3.5. Effects of Chemotherapy on Activating NK Cell Receptors

**Figure 6:**
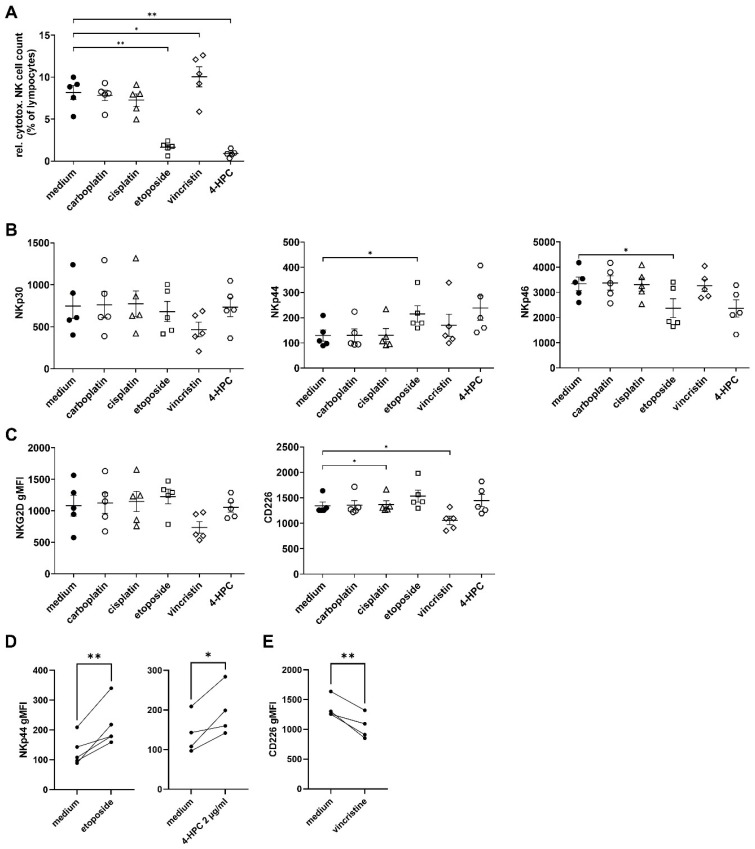
Impact of chemotherapy on percentage of (**A**) cytotoxic NK cells of lymphocytes and NK-cell-specific activating receptors. (**B**–**E**) 5 × 10^6^ PBMCs were treated for 24 h under cell culture conditions with either carboplatin (2 µg/mL, open circles), cisplatin (1 µg/mL, open triangles), etoposide (0.5 µg/mL, open squares), vincristine (0.05 µg/mL, open diamonds), or the cyclophosphamide metabolite (4-HPC, 1 µg/mL, open hexagons). After 72 h of culturing, cells were analyzed for NKp30, NKp44, NKp46, NKG2D, and CD226 expression, using flow cytometry. (**A**) Relative number of cytotoxic NK cells (CD3^−^, CD56^dim^) in lymphocytes. (**B**–**E**) Geometric mean fluorescence intensity (gMFI) of respective activating receptor of cytotoxic NK cells after chemotherapy. Data represent at least four biological replicates. Means and SEM are indicated as black lines and error bars, respectively. For statistical analysis, repeated measures ANOVA with appropriate post hoc test was used; * *p* < 0.05 vs., ** *p* < 0.01 versus untreated control (medium).

To further evaluate the immunological effects of chemotherapy on NK cells, we determined the percentage of cytotoxic NK cells (CD56^dim^) in lymphocytes and measured activating NK cell receptors (NKp30, NKp44, NKG2D, and CD226) for the reported stress ligands by flow cytometry. Etoposide and cyclophosphamide significantly reduced cytotoxic NK cell abundance compared to the medium control (1.64 ± 0.26%, 0.89 ± 0.17% vs. 8.17 ± 0.73% in live lymphocytes, respectively; see Figure 6A), whereas vincristine treatment significantly increased the NK-cell number (10.04 ± 1.7%, Figure 6A). Most chemotherapeutics did not affect stress-ligand receptors (Figure 6B,C). However, etoposide and cyclophosphamide significantly increased NKp44 expression (1.66- and 2.2-fold increase; see Figure 6B,D), whereas NKp46 and CD226 were significantly decreased by etoposide and vincristine treatment (1.41- and 1.47-fold decrease, respectively; see Figure 6B,C,E).

In conclusion, etoposide and cyclophosphamide increased the activating receptor NKp44, and vincristine increased the number of cytotoxic NK cells, indicating an immunological impact of etoposide, cyclophosphamide, and vincristine on NK cells.

### 3.6. Role of Stress Ligands in Chemotherapy-Mediated Antitumor Efficacy of the Anti-GD2 Treatment

**Figure 7:**
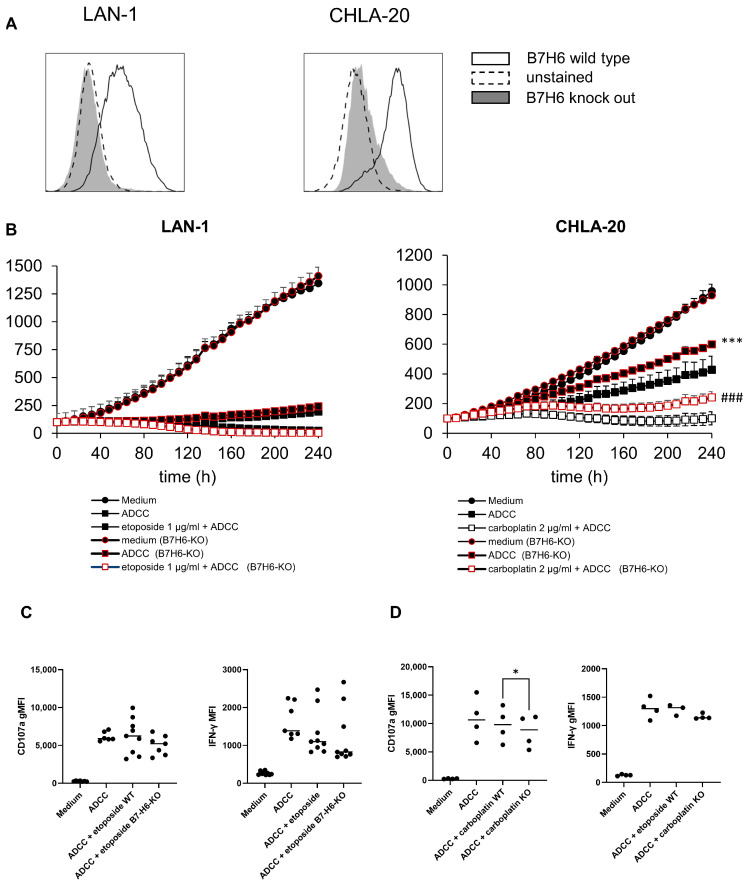
Role of chemotherapy-induced stress ligands in NK cell activation. B7H6 was deleted by using CRISPR/CAS9 system, and (**A**) successful knock out was confirmed by flow cytometry. The data represent means of at least three independent experiments. (**B**) Graphs show viability of spheroids treated with or without chemotherapy (black line, white or black marker, respectively), PBMCs alone (solid triangle), ADCC (solid square). Viability data for B7-H6-KO cells are shown with red bordered markers. (**C**,**D**) CD107a and IFN-γ degranulation assay was performed after 24 h chemotherapy with etoposide (**C**, LAN-1) and carboplatin (**D**, CHLA-20) and three days overall incubation. Then 5 × 10^6^ PBMCs were co-incubated with 1 × 10^6^ tumor cells, followed by flow cytometric analysis of NK cells (CD45^+^, CD^3−^, CD56^dim^), using degranulation (CD107a) and activation marker (IFN-γ). Difference between viability after 240 h ADCC (wild type) vs. ADCC (B7-H6-KO) and ADCC (wild type) + chemotherapy vs. ADCC + chemotherapy (B7-H6-KO) was assessed by using paired *t*-test. (**A**) *** *p* < 0.001 vs. ADCC and ^###^ *p* ≤ 0.001 vs. chemoimmunotherapy (wild type). (B) *** *p* < 0.001 vs. ADCC and ^###^ *p* < 0.001 vs. chemoimmunotherapy. (D) ^*^ *p*< 0.05 vs. chemoimmunotherapy (wild type).

B7-H6 stress ligand and NKp30 receptor engagement have been shown to play a crucial role in NK-cell activation. Therefore, we deleted the B7-H6 gene in LAN-1 and CHLA-20 cells to investigate the role of B7-H6 interaction in the cytotoxicity of chemoimmunotherapy in our model (Figure 7).

Since chemoimmunotherapy with carboplatin showed a strong effect compared to ADCC controls (Figure 2B) and carboplatin exclusively increased B7-H6 surface abundance (Figure 4A, center), we tested the hypothesis that a B7-H6 knockout (KO) in CHLA-20 cells will reverse some of the beneficial effects of the carboplatin-based chemoimmunotherapy. Additionally, we investigated the impact of a B7-H6-KO in LAN-1 cells treated with etoposide-based chemoimmunotherapy, as this was highly effective (Figure 3A), and etoposide treatment activated all stress ligands analyzed in wild-type LAN-1 cells (Figure 4A–C left panel).

Indeed, we found that the B7-H6-KO of CHLA-20 cells significantly reversed the chemoimmunotherapy effect of cisplatin, carboplatin and vincristine compared to the wildtype control (Figure 7B, right panel; and Supplementary Figure S1). Since the viability was also improved under ADCC conditions in B7-H6 KO-cells, the effect was mainly attributable to the B7-H6-KO.

In contrast, LAN-1 cells did not show any dependency on B7-H6 (Figure 7B left panel). In summary, we found a partial B7-H6-dependency in CHLA-20 but not in LAN-1 maybe due to strong checkpoint induction after chemoimmunotherapy (Figure 2A–D, left panel).

### 3.7. Antibody-Mediated NK Cell Activation

To further investigate whether checkpoint- and stress-ligand-induction affect NK cell activation, we measured the activation of NK cells by means of degranulation (CD107a) and IFN-γ production, using flow cytometry, as described in the Materials and Methods section. For that, we cultured the PBMCs of healthy donors for 5 h with DB and LAN-1- and CHLA-20 tumor cells (wild type and B7-H6-KO) pretreated with etoposide and carboplatin, respectively. The ADCC conditions showed a strong NK cell degranulation (CD107a) and activation (IFN-γ) (Figure 7).

The chemotherapy-treated tumor cells did not enhance NK cell activation compared to untreated LAN-1 and CHLA-20 cells (Figure 7C,D). Indeed, the activation of NK cells against LAN-1 with B7-H6-KO was markedly, but not significantly, decreased, and against CHLA-20, B7-H6-KO was significantly reduced (Figure 6C, *p* = 0.0831; and Figure 7D, *p* = 0.0265). Overall, we could not observe a stronger activation of NK cells by chemotherapy-treated compared to untreated tumor cells.

## 4. Discussion

We evaluated the effects of chemotherapeutics currently used in the standard induction regimen to treat patients with HR-NB in combination with the anti-GD2 antibody DB against spheroids generated from tumor cells derived from patients with progressive disease. Antitumor efficacy of chemoimmunotherapy was superior compared to the chemotherapy or DB in the presence of immune effector cells (ADCC) alone (up to 17-fold decrease in viability compared to ADCC; see Figure 5 and Figure 6 and Table 1). Our data provide preclinical proof-of-concept for a combined use of chemotherapy with anti-GD2 antibodies against neuroblastoma.

We developed a spheroid viability assay that allowed us to measure the long-term effects of the chemotherapeutic compound at clinically relevant concentrations (Figure 1). Live-cell microscopy using fluorescent tumor cells provides the advantage of undisturbed long-term viability analysis. Our approach circumvents the common problem of short-term viability assays that lead to EC50 values that are too high to be achieved in patients [22]. Additionally, a spheroid represents a model that is closer to the clinical reality compared to 2D models [26]. The architecture of a spheroid provides a nutrition and oxygen gradient that can result in the development of cancer-stem-like cells that represent a chemotherapy resistant subgroup of high clinical relevance [39]. However, this model can be further improved by incorporating multiple cell types, such as cancer-associated fibroblast and myeloid-derived suppressor cells to mimic an inhibitory tumor microenvironment [30]. The spheroid model used here is limited, as it does not reflect anti-angiogenic effects of chemotherapy and the role of fluidic shear stress in metastasis [28,40,41]. Despite these limitations, we have shown that a long-term spheroid viability assay is an appropriate tool to analyze combined effects of chemotherapy with antibody-dependent NK-cell-mediated tumor-cell lysis (Figure 2 and Figure 3).

NK cells are the main effector cells mediating the effect of DB, and the activation of NK cells depends on an equilibrium of inhibitory receptors [42], such as killer-cell immunoglobulin-like receptors (KIRs) and PD-1, as well as activating receptors, such as NKGD2 and NKp30 [15], binding to the stress ligands ULBPs and MICA/B, as well as B7-H6, respectively.

The induction of stress ligands on neuroblastoma cells (Figure 4) and, to a lesser extent, of activating receptors on NK cells (Figure 6) might explain the synergistic efficacy of the chemoimmunotherapy (Figure 2 and Figure 3). In line with that, it has been shown that B7-H6 sensitizes HEK293 cells for NK cell-mediated cytotoxicity [16]. Since NKp30 plays a crucial role in NK-cell activation and tumor surveillance, the increased expression of its cognate ligand B7-H6 by chemotherapeutics enhances NK cell-mediated ADCC against tumor cells [43].

However, we also found a chemotherapy-dependent induction of the checkpoint ligand expression on neuroblastoma, namely PD-L1, CD86, CD155, and Gal-9 (up to 5-fold increase vs. control; see Figure 5). Checkpoint ligand expression correlates with poor survival attributed to inhibition of immune surveillance [44]. This observation suggests that we consider checkpoint inhibitors in chemoimmunotherapy concepts. Another limiting aspect for chemoimmunotherapy may be the effects of chemotherapy on NK cell viability that might directly impact the efficacy of an ADCC-based immunotherapy. For instance, in a clinical study in acute lymphoblastic leukemia that also includes vincristine treatment, the total lymphocyte rate was reduced 18 months after maintenance chemotherapy [45]. Here, platin agents did not negatively affect cytotoxic NK cell count and vincristine even increased the NK cell to lymphocyte ratio (Figure 6A). In contrast, etoposide and cyclophosphamide reduced the number of cytotoxic NK cells (Figure 6A). However, etoposide and cyclophosphamide significantly increased the activating receptor NKp44 levels on cytotoxic NK-cells (Figure 6B,D). Importantly, NKp44, but not NKp30 and NKp46, is an activation marker for cytotoxic NK cells (Figure 6B,D) [46,47].

This underlines the ambiguous effect of chemotherapy with beneficial but also detrimental consequences for immunotherapy, which is dependent on a functional immune effector cells [48]. In light of the encouraging in vitro effects observed here, it remains crucial to evaluate this concept in patients.

In addition to effects of chemotherapy on NK cells and the role of immune checkpoint pathways, the inhibitory tumor microenvironment and inhibitory leukocyte populations have to be considered for a more comprehensive picture of a DB-based chemoimmunotherapy in HR-NB.

Despite a substantial increase of checkpoint ligands during chemotherapy (Figure 5), we could demonstrate that chemoimmunotherapy with DB improved efficacy in our models (Figure 2 and Figure 3). Regardless of the much weaker stress response in CHLA-136 spheroids compared to LAN-1 and CHLA-20 spheroids, chemoimmunotherapy with cisplatin and etoposide was more effective compared to the monotherapies. CHLA-136 spheroids showed higher resistance toward cyclophosphamide and vincristine, and, consequently, these agents did not further improve the ADCC effect.

Finally, we observed that ADCC was partially B7-H6-stress-ligand dependent (Figure 7B). However, we could not find evidence to support the hypothesis that chemotherapy-induced stress ligands improved ADCC (Figure 7C). This might be attributable to the observed strong induction of checkpoint ligand expression found in tumor cells after chemotherapy. Accordingly, we and others showed that PD-L1 was also elevated by ADCC and IFN-γ via JAK/STAT signaling [49]. On top of that, chemotherapy can increase NFκB-signaling, which, in turn, can elevate PD-L1 expression [50,51]. Intriguingly, NFκB is a transcription factor that also positively regulates GD3-synthase and, therefore, GD2-abundance in cancer stem cells [52]. Increased NFκB expression might therefore lead to higher GD2 abundance and increased susceptibility toward anti-GD2 treatment, which is subject to further research.

## 5. Conclusions

In conclusion, chemotherapy used in European chemotherapy induction regimens for HR-NB combined with antibody-based immunotherapy can effectively eradicate tumor spheroids derived from relapsed/refractory patients. Our results encourage the implementation of DB in the induction therapy in future clinical trials.

## Figures and Tables

**Table 1:** Overview of viability in %±SEM after 240 h of respective treatment. Viability was calculated as total integrated NIR intensity after 240 h divided by total integrated NIR intensity at 0 h. Statistical difference was assessed by using repeated measure ANOVA; ** *p* < 0.01 vs. and * *p* < 0.05 for ADCC + chemotherapy vs. AICC + chemotherapy.

		Viability ± SEM (240 h in %Compared to 0 h)					Fold Decrease in Viability of ADCC + Chemotherapy vs.			
Cell Line	Therapeutics	Medium	Chemo.	ADCC	AICC + Chemo.	ADCC + Chemo.	Chemo.	ADCC	AICC + Chemo.	*p*-Value
**LAN-1**	carboplatin	1120 ± 96	877 ± 128	352 ± 106	615 ± 150	199 ± 85	4.4	1.8	3.1	** 0.0075
	cisplatin	1222 ± 107	399 ± 62	302 ± 119	276 ± 52	84 ± 58	4.7	3.6	3.3	** 0.0059
	4-HPC	962 ± 127	503 ± 48	426 ± 126	434.7 ± 87	265 ± 65	1.9	1.6	1.6	* 0.0317
	etoposide	930 ± 53	146 ± 29	309 ± 59	113 ± 27	17 ± 8	8.3	17.6	6.4	* 0.0178
	vincristine	1211 ± 95	397 ± 91	352 ± 107	306 ± 90	115 ± 47	3.5	3.1	2.7	* 0.0337
**CHLA-20**	carboplatin	958 ± 47	330 ± 75	428 ± 47	449 ± 69	100 ± 44	3.3	4.2	4.5	* 0.0204
	cisplatin	938 ± 41	399 ± 66	460 ± 77	387 ± 69	138 ± 61	2.9	3.3	2.8	** 0.0097
	4-HPC	673 ± 41	408 ± 19	409 ± 66	319 ± 3.2	204 ± 6.4	2.0	2.0	1.6	* 0.0013
	etoposide	917 ± 40	762 ± 71	436 ± 67	587 ± 78	413 ± 49	1.8	1.1	1.4	0.0653
	vincristine	1039 ± 62	765 ± 77	479 ± 64	579 ± 50	261 ± 72	2.9	1.8	2.2	** 0.0040
**CHLA-136**	carboplatin	454 ± 33	348 ± 31	248 ± 64	228 ± 39	164 ± 43.6	2.1	1.5	1.4	** 0.0045
	cisplatin	415 ± 54	304 ± 31	192 ± 77	188 ± 100	93 ± 46	3.3	2.1	2.0	0.1796
	4-HPC	334 ± 53	327 ± 26	100 ± 67	93 ± 48	110 ± 57	0.9	1.0	0.8	0.9215
	etoposide	400 ± 24	203 ± 16.4	227 ± 52	105 ± 15	54 ± 19.6	3.7	4.6	2.9	0.0954
	vincristine	414 ± 34	358 ± 33	305 ± 77	233 ± 24	230 ± 42	1.6	1.1	1.0	0.9995

## Data Availability

The data that support the findings of this study are available from the corresponding author (H.L) upon reasonable request.

## Supplementary Materials

The following supporting information can be downloaded at https://www.mdpi.com/article/10.3390/cancers15030904/s1. Figure S1: B7-H6-KO results for CHLA-20. Video S1: Spheroid viability medium control. Video S2: Spheroid viability during cisplatin chemotherapy. Video S3: Spheroid viability during chemoimmunotherapy (cisplatin + ADCC).
