# Supplementary figures and images for: Chemotherapeutics Used for High-Risk Neuroblastoma Therapy Improve the Efficacy of Anti-GD2 Antibody Dinutuximab Beta in Preclinical Spheroid Models
Source: Cancers (Basel). 2023 Jan 31;15(3):904. doi: 10.3390/cancers15030904 (PMC9913527; doi:10.3390/cancers15030904)

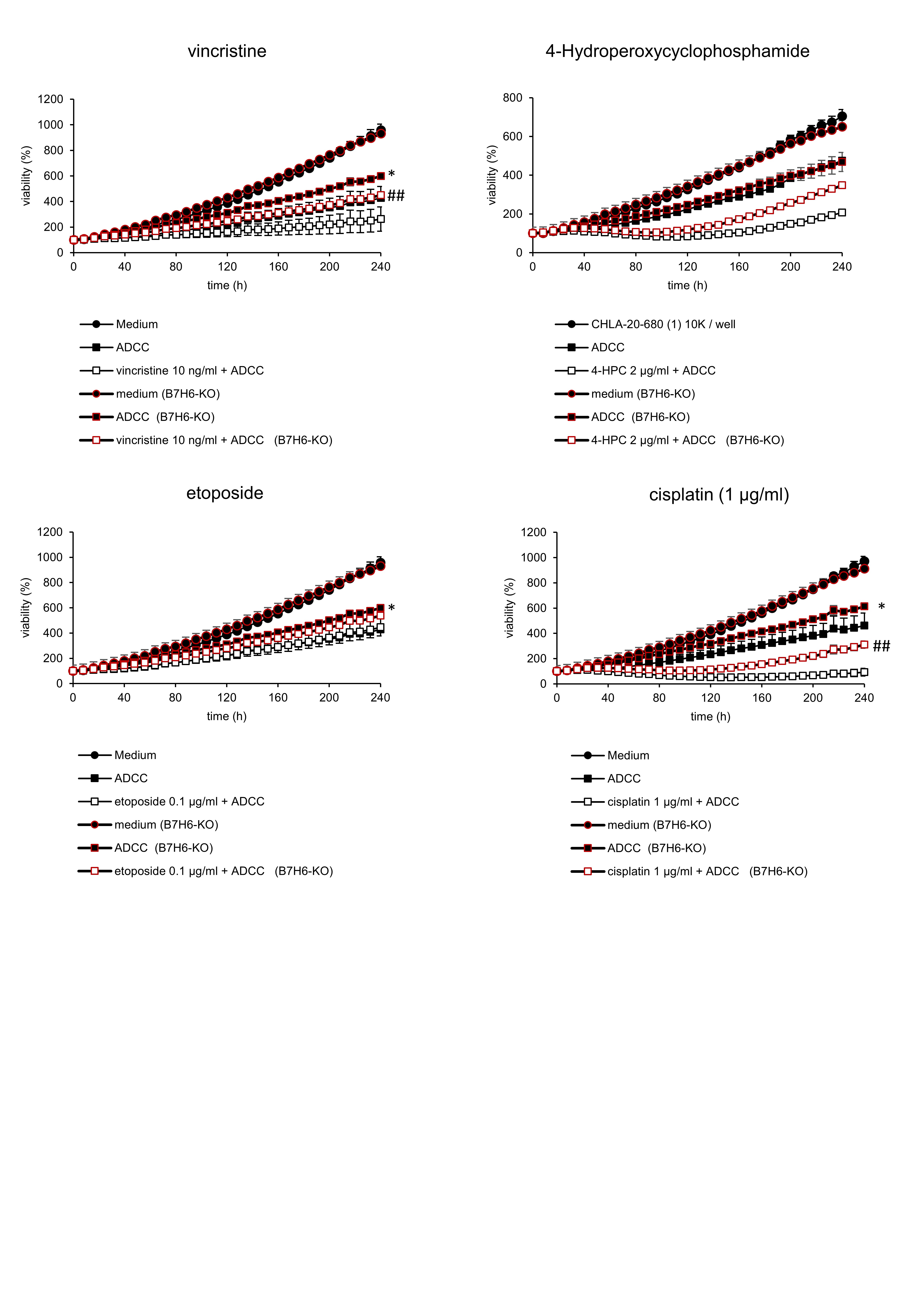

Supplement: Supplementary file 1 [file cancers-15-00904-s001.zip › suppl. Figure S1.JPG]
